# Enhanced transcriptomic responses in the Pacific salmon louse *Lepeophtheirus salmonis oncorhynchi* to the non-native Atlantic Salmon *Salmo salar* suggests increased parasite fitness

**DOI:** 10.1186/s12864-017-3520-1

**Published:** 2017-01-30

**Authors:** Laura M. Braden, Ben J. G. Sutherland, Ben F. Koop, Simon R. M. Jones

**Affiliations:** 1 Centre for Biomedical Research, University of Victoria, Victoria, British Columbia Canada; 2 Pacific Biological Station, Fisheries & Oceans Canada, Nanaimo, British Columbia Canada; 3 Present Address: Department of Pathology and Microbiology, Atlantic Veterinary College, Charlottetown, Prince Edward Island Canada; 4 Present Address: Département de biologie, Institut de Biologie Intégrative et des Systèmes (IBIS), Université Laval, Québec, Québec Canada

**Keywords:** Copepoda, Host susceptibility, *Lepeophtheirus salmonis*, Resistance, Salmon, Sea lice, Virulence factors

## Abstract

**Background:**

Outcomes of infections with the salmon louse *Lepeophtheirus salmonis* vary considerably among its natural hosts (*Salmo, Oncorhynchus* spp.). Host-parasite interactions range from weak to strong host responses accompanied by high to low parasite abundances, respectively. Parasite behavioral studies indicate that the louse prefers the host Atlantic Salmon (*Salmo salar*), which is characterized by a weak immune response, and that this results in enhanced parasite reproduction and growth rates. Furthermore, parasite-derived immunosuppressive molecules (e.g., proteases) have been detected at higher amounts in response to the mucus of Atlantic Salmon relative to Coho Salmon (*Oncorhynchus kisutch*). However, the host-specific responses of the salmon louse have not been well characterized in either of the genetically distinct sub-species that occur in the Atlantic and Pacific Oceans.

**Results:**

We assessed and compared the transcriptomic feeding response of the Pacific salmon louse (*L. salmonis oncorhynchi,*) while parasitizing the highly susceptible Atlantic Salmon and Sockeye Salmon (*Oncorhynchus nerka*) or the more resistant Coho Salmon (*Oncorhynchus kisutch*) using a 38 K oligonucleotide microarray. The response of the louse was enhanced both in the number of overexpressed genes and in the magnitude of expression while feeding on the non-native Atlantic Salmon, compared to either Coho or Sockeye Salmon. For example, putative virulence factors (e.g., *cathepsin L, trypsin, carboxypeptidase B*), metabolic enzymes (e.g., *cytochrome B, cytochrome C*), protein synthesis enzymes (e.g., *ribosomal protein P2, 60S ribosomal protein L7*), and reproduction-related genes (e.g., *estrogen sulfotransferase*) were overexpressed in Atlantic-fed lice, indicating heightened parasite fitness with this host species. In contrast, responses in Coho- or Sockeye-fed lice were more similar to those of parasites deprived of a host. To test for host acclimation by the parasite, we performed a reciprocal host transfer experiment and determined that the exaggerated response to Atlantic Salmon was independent of the initial host species, confirming our conclusion that the Pacific salmon louse exhibits an enhanced response to Atlantic Salmon.

**Conclusions:**

This study characterized global transcriptomic responses of Pacific salmon lice during infection of susceptible and resistant hosts. Similar parasite responses during infection of Coho or Sockeye Salmon, despite differences in natural immunity to infection between these host species, indicate that host susceptibility status alone does not drive the parasite response. We identified an enhanced louse response after feeding on Atlantic Salmon, characterized by up-regulation of virulence factors, energy metabolism and reproductive-associated transcripts. In contrast, the responses of lice infecting Coho or Sockeye Salmon were weaker, with reduced expression of virulence factors. These observations indicate that the response of the louse is independent of host susceptibility and suggest that co-evolutionary host-parasite relationships may influence contemporary host-parasite interactions. This research improves our understanding of the susceptibility of Atlantic Salmon and may assist in the development of novel control measures against the salmon louse.

**Electronic supplementary material:**

The online version of this article (doi:10.1186/s12864-017-3520-1) contains supplementary material, which is available to authorized users.

## Background

In a host–parasite relationship at equilibrium, parasite fitness is optimized and costs to the host are minimized. Disequilibrium of this relationship can cause harm to the host in the case of heightened virulence [1], or conversely, rejection of the parasite caused by heightened host immunity [2]. Aggressive host environments (e.g. a robust immune response) have been shown to decrease parasite fitness. For example, reduced reproductive output in ticks (*Rhipicephalus microplus*) is associated with feeding on more resistant hosts [3]. Increased parasite fitness while parasitizing more susceptible hosts is also observed in the differential developmental rates of the parasitic copepods *Caligus rogercresseyi* and *Lepeophtheirus salmonis* while infecting susceptible species (*Oncorhynchus mykiss* and *Salmo salar*, respectively), compared with that of resistant species [4, 5]. In addition, fewer low molecular weight proteases (virulence factors) are secreted by *L. salmonis* in response to mucus from resistant salmon (*O. kisutch*) relative to that from more susceptible hosts such as *S. salar* or *O. mykiss* [6] indicating that in addition to reducing reproductive output and development rate, an aggressive host response may also interfere with physiological responses associated with parasite feeding.

The salmon louse, *L. salmonis*, is a naturally occurring parasitic copepod that parasitizes anadromous salmonids belonging to the genera *Oncorhynchus* and *Salmo* spp, and is consequently an important pest of salmonid mariculture [7–9] throughout the Northern Hemisphere with sub-species in the Pacific (*L. salmonis oncorhynchi*) and Atlantic (*L. salmonis salmonis*) Oceans [10]. During heavy infestations, degradation of the epidermis and mucosal layer leads to osmoregulatory distress, anaemia, lethargy, secondary infections and a general stress response [11–13]. Resistance to the parasite varies among juvenile salmon such that Coho Salmon (*Oncorhynchus kisutch*) [14, 15] and Pink Salmon (*Oncorhynchus gorbuscha*) [16–18] display a resistant phenotype characterized by well-developed inflammation at the attachment site, rapid parasite rejection and limited additional pathology. In contrast, Atlantic Salmon (*S. salar*) [15, 17–21], Chum Salmon (*Oncorhynchus keta*) [16–18], and Sockeye Salmon (*Oncorhynchus nerka*) [15, 22] display a susceptible phenotype characterized by weak or absent local inflammation, higher parasite burden and associated pathology.

The differing host responses to the salmon louse suggest that there are also host-specific parasite responses. Behavioural studies indicate a preference of *L. salmonis* for salmonid over non-salmonid hosts [23, 24], and in particular a preference for Atlantic Salmon [25, 26]. Secretions from *L. salmonis* elicited by Atlantic Salmon mucus or dopamine extraction contain prostaglandin E_2_ (PGE_2_) and trypsin proteases, which are potent immune-modulators [27]. However, there are many questions that remain including the mechanisms involved in the differential host responses, and whether the secretion of virulence factors is influenced by the host species. Furthermore, most of the foundational studies on the differences in susceptibility in hosts (described above) have not considered the co-evolutionary history of the host-parasite relationship.

There is a significant knowledge gap regarding the response of the salmon louse during feeding. To this end, we applied a transcriptomic approach to assess the effect of host resistance on louse feeding responses. We hypothesized that the transcriptomic feeding response on more susceptible species (Atlantic, Sockeye Salmon) would be enhanced in virulence factors and fitness-related pathways (e.g. feeding, reproduction, energy metabolism) relative to that elicited by a resistant species (Coho Salmon). However, our results indicate a specific enhanced response to Atlantic Salmon that was not explained by host acclimation but that may be due to differences in co-evolutionary history of the parasite and the hosts.

## Results

**Fig. 1:**
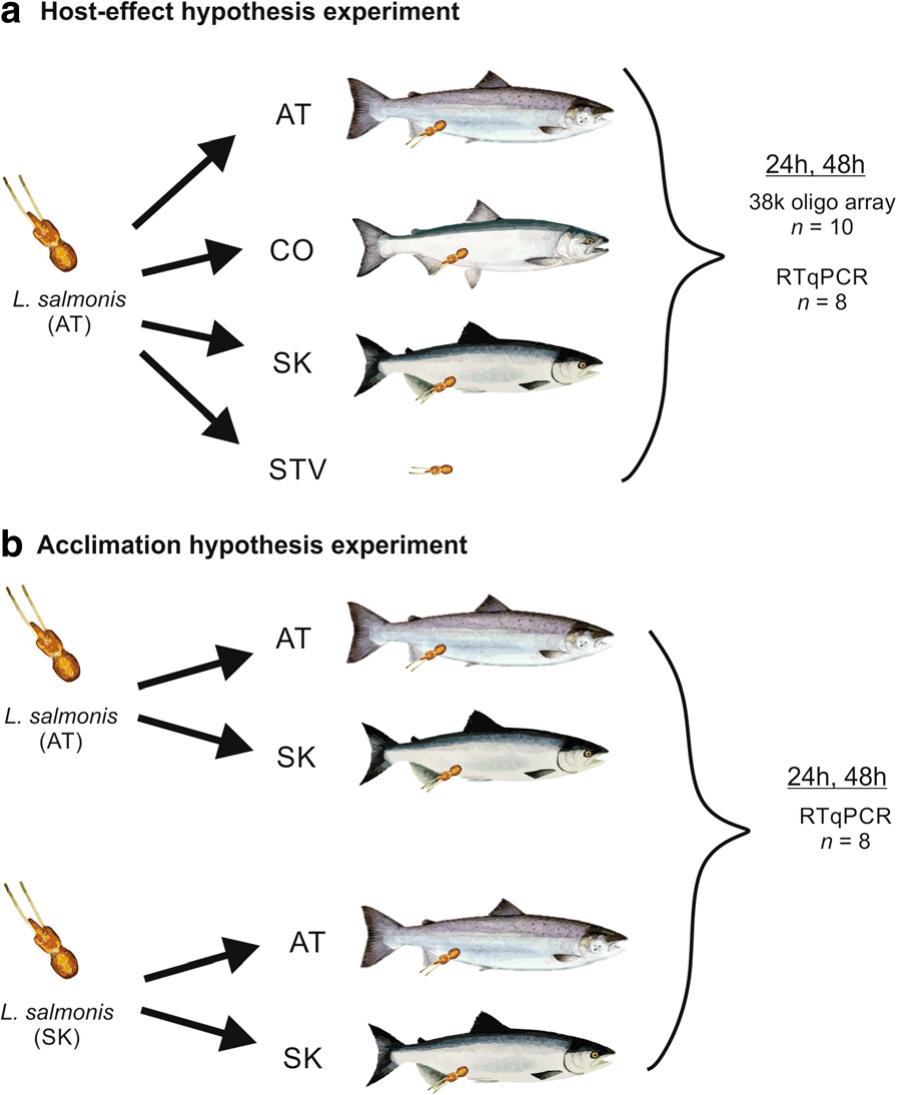
Experimental design. In the host-effect hypothesis experiment (**a**) Atlantic (AT), Coho (CO), and Sockeye (SK) Salmon were infected with *L. salmonis* sourced during commercial Atlantic Salmon harvest. A sub-set of unattached lice served as the starved (STV) control. At 24 and 48 h lice (*n* = 10) were removed from every species and processed for down-stream microarray and RT-qPCR analysis. In the acclimation hypothesis experiment (**b**) *L. salmonis* were sourced from Atlantic Salmon (AT) during harvest as well as from Sockeye Salmon (SK) from a test fishery. Lice from each species (AT, SK) were used to infect both Atlantic and Sockeye Salmon. At 24 and 48 h, lice (*n* = 8) were removed and processed for downstream RT-qPCR analysis

Using a *L. salmonis* 38 K oligonucleotide microarray (eArray, Agilent) designed with expressed sequence tags (ESTs) from *L. salmonis salmonis* and *L. salmonis oncorhynchi* [28], we detected 15,718 probes that passed quality control filters. Excluding duplicate probes, this included 8,776 unique transcripts. Differences in the expression of these genes were investigated *L. salmonis* infecting Atlantic, Coho, or Sockeye Salmon and in lice withheld from a host (i.e., starved; Fig. 1a).

### Profiling the feeding response of the salmon louse

**Fig. 2:**
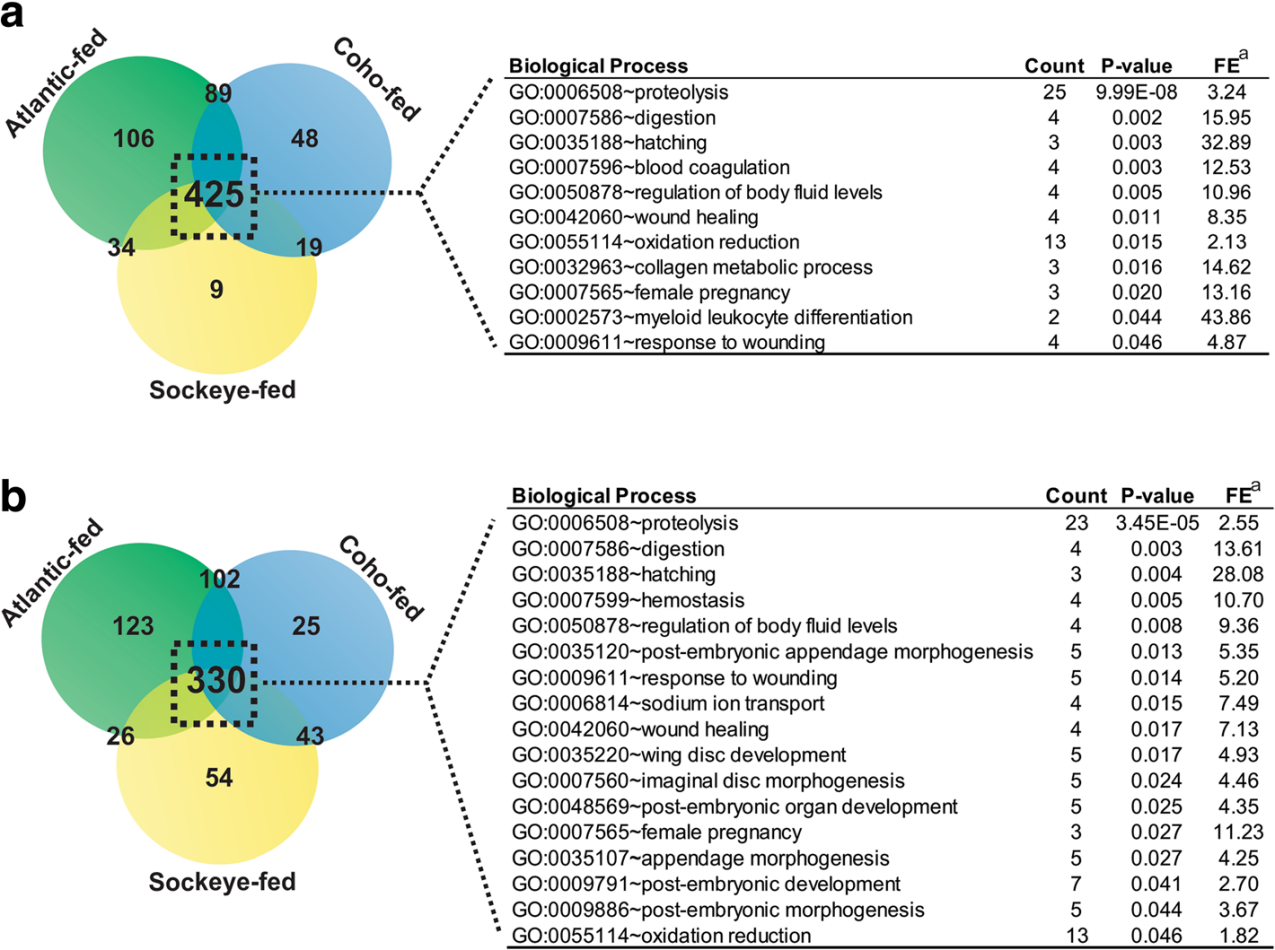
Profiling the feeding response of *L. salmonis.* Overexpressed transcripts in lice feeding on Atlantic, Coho or Sockeye Salmon relative to starved lice were compared to produce a list of unique genes involved in the feeding response of *L. salmonis*. These genes were analyzed using DAVID to produce GO enrichment results after **a** 24 and **b** 48 hpi. ^a^Fold Enrichment

The total number of differentially expressed genes (DEGs) at 24 and 48 hpi in lice parasitizing each host (Atlantic, Coho, and Sockeye Salmon) was compared to a group of lice withheld from hosts (i.e., ‘starved’; Additional file 1: Table S1). The feeding response of *L. salmonis* was determined by identifying DEGs common to lice parasitizing salmon regardless of species (*n* = 10 individual lice per host species per time point; total = 60 feeding lice and 20 starved). Gene Ontology analysis of overexpressed feeding DEGs revealed enrichment in biological processes such as proteolysis (GO:0006508), hatching (GO:0035188), blood coagulation (GO:0007596), oxidation reduction (GO:0055114), and collagen metabolic process (GO:0032963) (Fig. 2).

Using RPS-BLAST and the Conserved Domain Database [29] as previously described [30], conserved domains were identified within the feeding response genes. This analysis revealed 67 distinct domains overexpressed at 24 hpi with frequently identified domains including “trypsin-like” (29 unique contigs, smart00020), “saposin-like” (7 unique contigs, smart00741), and “cysteine-like” (4 unique contigs, cd03860). At 48 hpi there were 71 distinct domains, with the most frequently identified domains including “trypsin-like” (19 unique contigs, smart00020) and “glutathione peroxidase-like” (7 unique contigs, cd00340). As expected, these domains are associated with feeding and digestion. The “trypsin-like serine protease” domain (smart00020, cd00190) was present in 14 genes including *trypsin-1, collagenase, chymotrypsin B1*, *coagulation factor IX* and *hypodermin B*. Also well represented were “peptidase” domains, including “peptidase C1” (e.g., *cathepsin L*; pfam00112), “peptidase C13” (e.g., *legumain*; pfam01650), “peptidase S28” (e.g., *putative serine protease K12H4.7*; pfam05577), “peptidase M13” (e.g., *neprilysin-2*; pfam01431), and “peptidase M14” (e.g., *carboxypeptidase B*; cd03860) (Additional file 2).

**Fig. 3:**
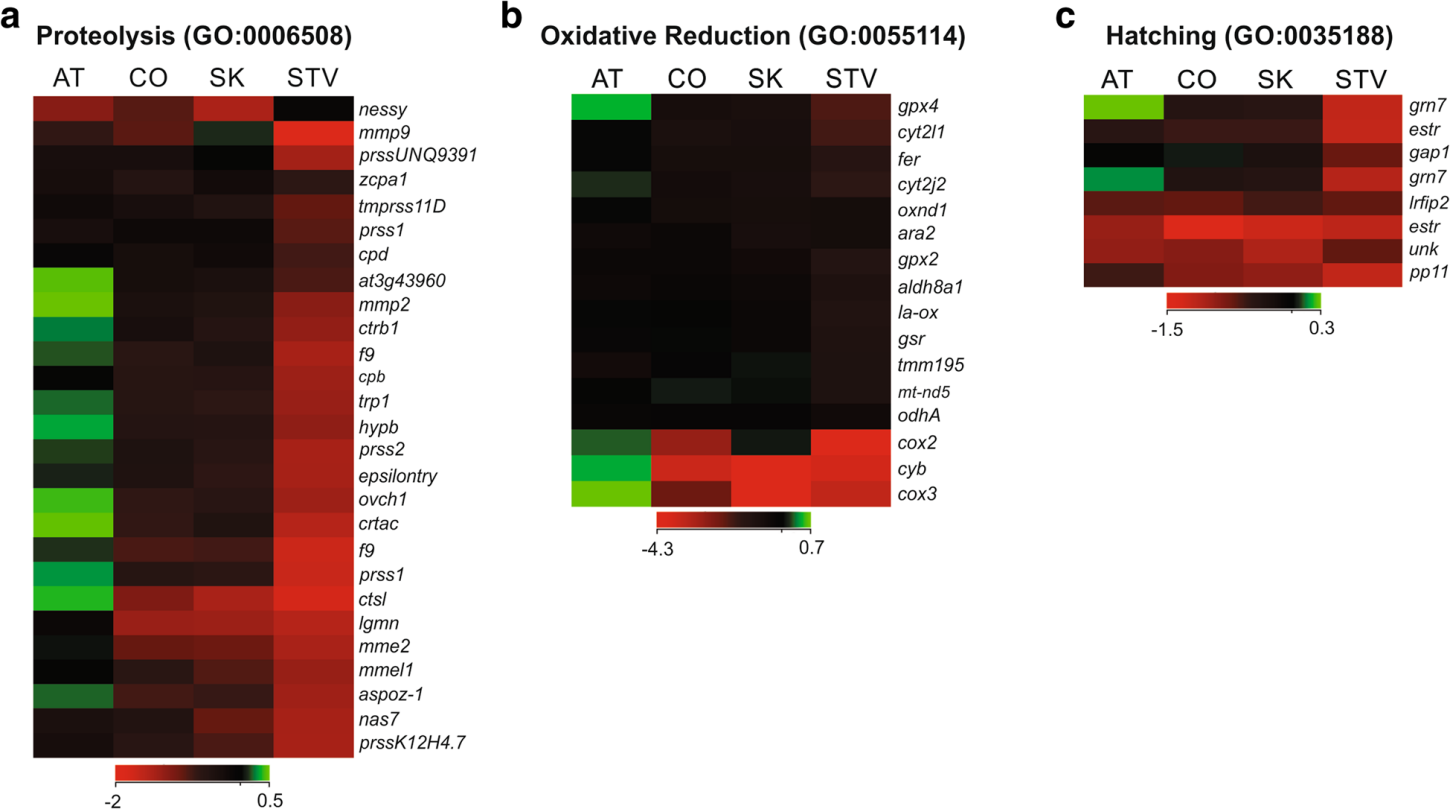
Enhanced transcriptomic response in Atlantic-fed lice. Hierarchical clustering of transcripts at 48 hpi enriched in GO biological process categories of **a** proteolysis, **b** oxidative reduction, and **c** hatching revealed an enhanced response specific to *L. salmonis* feeding on Atlantic Salmon (AT). Expression levels are mean log2 normalized intensities of each transcript, with red and green representing low and high expression, respectively. These genes were significantly differentially expressed in response to feeding on Atlantic Salmon compared to starved (*p* < 0.01; FC ≥ 1.5)

Although expression of these transcripts was evident during feeding on salmon regardless of species, Atlantic-fed lice exhibited the highest expression of proteolytic-, metabolic- (oxidative-reduction), and reproductive-associated (hatching) genes (Fig. 3).

Some specific transcripts were identified in Atlantic-fed lice that may be related to virulence in the salmon louse. For example, we detected a “phospholipase A2 bee venom-like” domain (cd04704) that was overexpressed at 48 hpi in Atlantic-fed lice compared to either Coho- or Sockeye-fed lice (FC = 2.03, 2.38, respectively). Additionally, “saposin B-like” domains (smart00741), known to be important in lipid-interacting proteins such as NK-lysins, were well represented on the array (e.g., 12 contigs passing fold-change filters at 48 hpi), and expression of this gene was significantly higher in Atlantic-fed lice (FC = 2.57–5.31, *p* < 0.001) compared to Coho-fed lice (FC = 1.5–1.94, *p* < 0.001), while Sockeye-fed lice expression of these domains did not pass the fold-change filter.

### Profiling the starvation response of the salmon louse

To determine the response to starvation, we evaluated overexpressed transcripts in starved lice compared to lice feeding on salmon, regardless of species. DEGs overexpressed in starved relative to salmon-fed lice represented the starvation response (Additional file 3: Figure S1). There were 47 and 143 transcripts overexpressed in starved *L. salmonis* at 24 and 48 h, respectively. Gene Ontology analysis of these unique “starved” DEGs revealed one significantly enriched category at 24 h (GO:0007165, signal transduction), and nine categories at 48 h including negative regulation of transcription (GO:0045892), cell differentiation (GO:0030154), and sarcomere organization (GO:0045214) (Additional file 3: Figure S1).

### Host-specific feeding responses of the salmon louse

We then assessed the host-specific responses of *L. salmonis* by focusing on the DEGs with FC ≥ 1.5 in Atlantic- compared to either Coho- or Sockeye-fed lice. In Atlantic-fed lice, an enhanced response was identified with an enrichment for digestion, reproduction and energy metabolism. Conversely in either Coho- or Sockeye-fed lice, the response was enriched for skeletal and sensory system development (Sockeye Salmon), or cytoskeletal organization, regulation of growth and nucleosome organization (Coho Salmon) (Additional file 4: Table S2).

At 24 hpi the most significantly enriched GO category in Atlantic-fed lice (*p* = 6.5 × 10^−4^ compared to Coho, *p* = 3.6 × 10^−9^ compared to Sockeye) was proteolysis (GO:0006508), and included proteases (*cathepsin L*, *trypsin-1*, *chymotrypsin A chain C, aspartic proteinase oryzasin-1, anionic trypsin-1*), carboxypeptidases (*carboxypeptidase B, zinc carboxypeptidase A1*), and matrix metalloproteases (*matrix metalloprotease 2, zinc metalloprotease nas-4*) (Table 1). In Coho-fed lice, the most significantly enriched GO category (*p* = 0.013 compared to Atlantic) was cytoskeletal organization (GO:0007010) and included genes like *troponin C isoform 1,* and *myosin heavy chain.* In Sockeye-fed lice, the most significantly enriched GO category (*p* = 0.006 compared to Atlantic) was skeletal system development (GO:0001501) and included genes like *zinc finger protein 16,* and *bone morphogenetic protein 2-B* (Additional file 4: Table S2).

**Table 1:** Proteolytic genes specific to Atlantic-fed lice

Gene	Unique contigs^a^	Accession	CDD	Fold change^b^
*Carboxypeptidase B*	5	P04069	cd03860	1.5, 4.1
*Chymotrypsin A chain C*	*1*	P00766	smart00020	4.4, 4.7
*Chymotrypsin BI*	*1*	Q00871	smart00020	2.7, 3.1
*Coagulation factor IX*	*1*	P16291	smart00020	1.7, 2.1
*Collagenase*	*1*	P08897	smart00020	1.8, 2.2
*Cytosolic non-specific dipeptidase*	*3*	Q96KP4	pfam01546	1.9, 2.1
*Dipeptidyl peptidase 4*	*1*	P14740	pfam00326	1.6, 2.8
*Hypodermin-B*	*1*	P35588	smart00020	2.2, 3.5
*Legumain*	*1*	Q4R4T8	pfam01650	1.8, 2.8
*Neprilysin-2*	*2*	O16796	pfam01431	3.0, 3.0
*Ovochymase-1*	*1*	Q7RTY7	smart00020	2.7, 5.9
*Probable cysteine proteinase At3g43960*	*1*	Q9LXW3	pfam00112	2.4, 2.6
*Putative serine protease K12H4.7*	*2*	P34528	pfam05577	1.9, 2.5
*Trypsin-1*	*5*	P00765	smart00020	1.6, 4.5
*Trypsin-like serine protease*	*2*	-	smart00020	1.8, 1.9
*Zinc carboxypeptidase A 1*	*3*	Q9VL86	cd03860	1.8, 2.3

Proteases that were significantly upregulated in Atlantic-fed lice compared to either Coho- or Sockeye-fed lice are shown (FC ≥ 1.5)

^a^Genes with similar annotation but from a different contig

^b^Compared to Pacific salmon (Coho and Sockeye, respectively) at 48 hpi

At 48 hpi, there was no significant enrichment of biological processes in Coho- or Sockeye-fed lice, whereas enriched pathways remained abundant and highly populated in Atlantic-fed lice. Some of the most pronounced overexpression specific to Atlantic-fed lice was observed in the genes involved in energy metabolism and protein synthesis, including genes enriched in the GO category oxidative reduction (GO:0055114), including *cytochrome C oxidase subunit 2* (FC = 4.1–8.4 compared to Coho-fed) and *3* (FC = 6.7–7.3 and 24.1–33.2, compared to Coho- and Sockeye-fed, respectively), and *cytochrome B* (FC = 19.8–32.3, 37.7–41.8, compared to Coho- and Sockeye-fed, respectively). Additionally, genes associated with protein synthesis such as *ribosomal protein P2* and *60S ribosomal protein L7,* were significantly overexpressed in Atlantic-fed lice (FC = 7.0–8.1 and 5.7, compared to Coho-fed lice, respectively; FC = 6.7–8.6 and 3.6, compared to Sockeye-fed lice, respectively) (Additional file 2).

Enrichment of genes associated with reproductive-type processes was also specific to Atlantic-fed lice and included the biological process GO categories of hatching (GO:0035188), female pregnancy (GO:0007565), blastocyst development (GO:0001824) and blastocyst hatching (GO:0001835). These categories were enriched in the response to Atlantic Salmon but not to either Coho or Sockeye Salmon, and included genes such as *placental protein 11, neutral ceramidase, granulin-7,* and *estrogen sulfotransferase* (Additional file 4: Table S2).

**Fig. 4:**
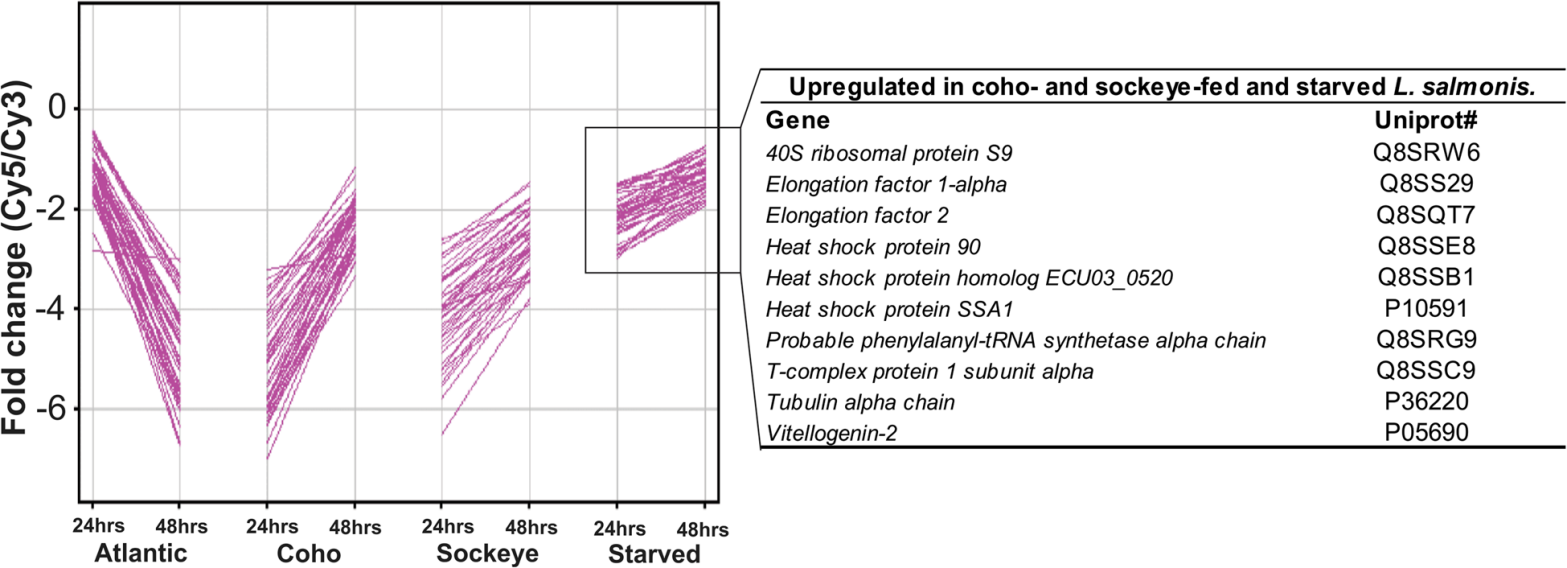
Similar expression profiles over time of stress-related transcripts in Coho-fed, Sockeye-fed and starved *L. salmonis*. Using *k-*means clustering analysis, genes associated with stress were shown to be up-regulated over time in lice feeding on Coho and Sockeye Salmon, and by lice withheld from hosts (Starved). In contrast, these genes were down-regulated over time in lice feeding on Atlantic Salmon

**Fig. 5:**
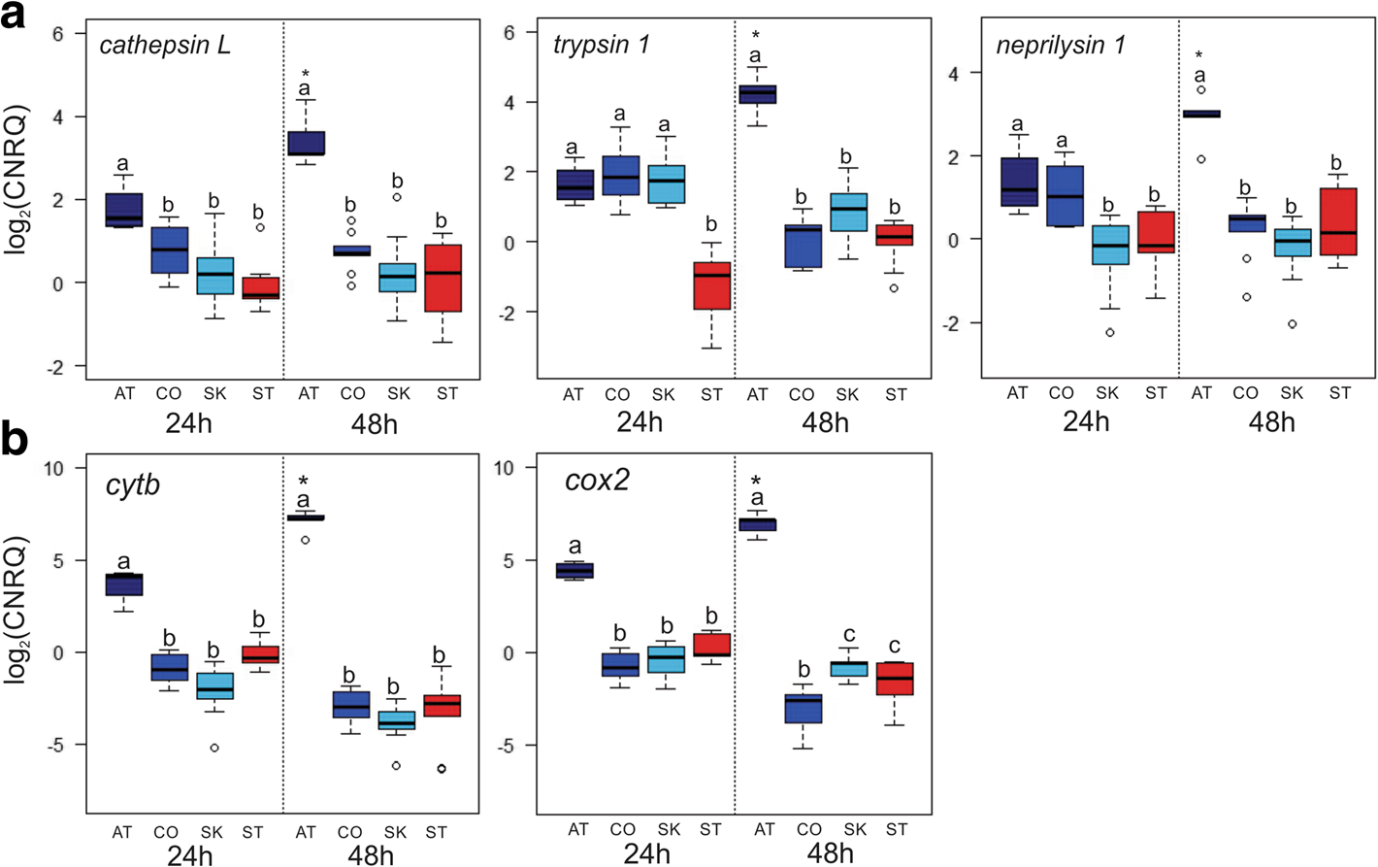
Enhanced expression of feeding and energy in Atlantic-fed lice. Differentially expressed transcripts identified by the microarray were profiled using RT-qPCR. Expression of genes involved in proteolysis/digestion (**a**) and energy metabolism (**b**) are shown as log2 calibrated normalized relative quantities (CNRQ). Expression in Atlantic-fed lice (AT) increased over time (24 → 48 hpi) and was significantly higher than in Coho-fed (CO) or Sockeye-fed (SK) lice. Expression of SK-fed and CO-fed lice was not significantly different from starved lice (ST). Significance was identified by two-way ANOVA (*p* < 0.05) followed by *post-hoc* Tukey HSD test to determine pairwise significance. Within a time point, lower-case letters denote differences between groups where groups that do not share a letter are significantly different. Asterisks denote differences within a group between time points

To characterize genes responding differently over time during infection with each species, *k*-means clustering of DEGs was performed and this further indicated the responses of Sockeye- and Coho-fed lice were similar to the starved lice and distinct from the Atlantic-fed lice. In one of the five clusters, 45 transcripts associated with stress, including *heat shock protein 90*, *T-complex protein 1 subunit alpha*, *heat shock protein SSA1*, and *heat shock protein homolog ECU03_0520* were up-regulated over time in Coho- and Sockeye-fed lice while down-regulated in Atlantic-fed lice (Fig. 4). Transcripts involved in feeding, energy metabolism and reproduction were most highly expressed while feeding on Atlantic Salmon (Figs. 3 and 5). Thus the feeding response of *L. salmonis* indicated that Atlantic Salmon is the most desirable host compared Coho or Sockeye Salmon.

### Temporal activation of the feeding response

We hypothesized that while parasitizing a more optimal host, responses associated with increased fitness (i.e., digestion, energy, reproduction) would increase over time (i.e. 24 → 48 hpi). The responses of Atlantic-fed lice increased over time, measured by the number of DEGs and the magnitude of expression of the DEGs. In contrast, the response in Coho-fed or Sockeye-fed lice either did not change, or was reduced. There was significant enrichment of up-regulated genes in the biological process categories such as proteolysis, digestion, and oxidative reduction in Atlantic-fed lice while enrichment of down-regulated transcripts was only observed in Coho-fed lice. There was no enrichment for up- or down-regulation over time in Sockeye-fed lice (Table 2).

**Table 2:** Enrichment in genes over time (24 → 48 hpi) in feeding *L. salmonis*

Biological Process	# genes	*p*-value	FE^a^
Increasing over time - Atlantic-fed *L. salmonis*			
GO:0055114 ~ oxidation reduction	9	0.005	3.1
GO:0019748 ~ secondary metabolic process	4	0.005	10.6
GO:0006769 ~ nicotinamide metabolic process	3	0.023	12.1
GO:0009820 ~ alkaloid metabolic process	3	0.023	12.1
GO:0010817 ~ regulation of hormone levels	3	0.023	12.1
GO:0019362 ~ pyridine nucleotide metabolic process	3	0.025	11.6
GO:0001501 ~ skeletal system development	3	0.048	8.2
GO:0019563 ~ glycerol catabolic process	2	0.021	93.0
GO:0046168 ~ glycerol-3-phosphate catabolic process	2	0.021	93.0
Decreasing over time - Coho-fed *L. salmonis*			
GO:0006979 ~ response to oxidative stress	3	0.009	18.8
GO:0006508 ~ proteolysis	5	0.046	3.2

^a^Fold enrichment

Conserved domain analysis was used to characterize the types of genes that were significantly different over time. Of the 435 genes affected by time (main effect time, no interaction effect), 211 were unannotated, 198 were annotated with domains, while 26 were annotated but did not contain a conserved domain. In total, 99 domains were present in the genes significantly overexpressed by *L. salmonis* over time (Additional file 2). Most domains were only represented by one gene, although 7 genes contained “trypsin-like serine protease” (smart00020, cd00190) domains, and 2 contained “peptidase M14 carboxypeptidase subfamily A/B-like*”* (cd03860), “FKBP-type peptidyl-prolyl cis-trans isomerase” (pfam00254), and “fasciclin” (pfam02469) domains. Genes with “trypsin-like serine protease” domains included *trypsin-1*, *ovochymase*-*2*, *neurotrypsin*, *chymotrypsin A*, and *trypsin-like serine protease*. Importantly, although these genes changed over time and contained similar protein domains, they did not change in the same way. For example, expression of *trypsin-1* increased over time in Atlantic-fed lice, and either declined or remained constant in Coho- or Sockeye-fed lice. In contrast, expression of *ovochymase-2* increased in Sockeye-fed lice, but remained constant in Atlantic- or Coho-fed lice.

Positively correlated expression profiles were observed for genes containing other domains. For example, genes with the “metallocarboxypeptidase” domain (cd03860; *carboxypeptidase B* and *zinc carboxypeptidase A1*) were both down-regulated over time in Coho-fed lice, and although expression in Sockeye-fed lice did not pass fold-change filters, there was a trend towards negative regulation for both these genes (FC = −1.4, −1.3, respectively).

Several other domains associated with proteolytic-enzymes were up-regulated over time only in Atlantic-fed lice including “peptidase M14 carboxypeptidase subfamily N/E-like” (cd03868), “zinc-dependent metalloprotease astacin-like subfamily” (cd04280), “papain family cysteine protease” (pfam00112), and “serine carboxypeptidase S28” (pfam05577).

One gene, *tyrosine aminotransferase* (TIGR01265), was concordantly down-regulated over time in Atlantic- (FC = −4.0), Coho- (FC = −1.6) or Sockeye-fed lice (FC = −2.0), indicating this gene is involved in the starvation stress response. Similarly, the “alpha crystallin” domain (cd06464; *programmed cell death 4*) was specifically associated with starvation.

### Enhanced transcriptomic response to Atlantic Salmon is not explained by acclimation

We considered the possibility that enhanced transcriptomic responses in Atlantic-fed lice were explained by acclimation to the host species, as the parasites had been collected from Atlantic Salmon. In a follow-up experiment, we assessed the expression of a subset of genes identified from the microarray experiment described above by exposing Atlantic and Sockeye Salmon to lice collected from either Atlantic or Sockeye Salmon (Fig. 1b). In the event of acclimation, we predicted enhanced responses in lice attached to salmon belonging to the same species as the source host. Contrary to this, our results showed enhanced responses of Atlantic-fed lice regardless of the source host species, for genes associated with protein synthesis (*ribosomal protein L2*, *60S ribosomal protein L7;* Additional file 5: Figure S2A), metabolism (*cytochrome oxidase subunit 2, cytochrome B;* Additional file *5* *:* Figure S2B), and proteolysis (*cathepsin L*; Additional file 5: Figure S2C). This indicates that the observed trends in expression were not due to acclimation, but rather to host-specific factors.

### Validation of the microarray

**Fig. 6:**
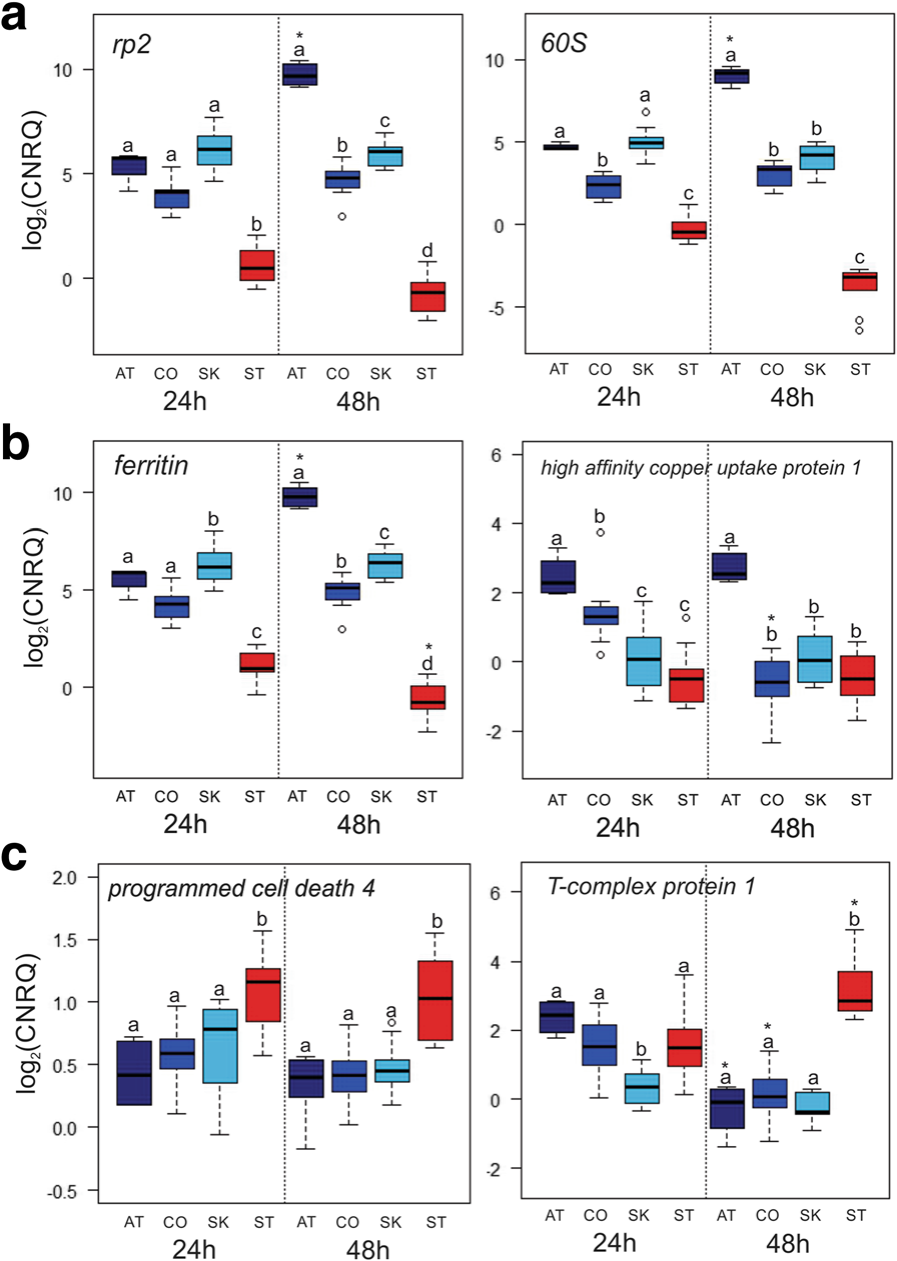
Enhanced expression of protein synthesis and oxidative-stress genes in Atlantic-fed lice. Differentially expressed transcripts identified by the microarray were profiled using RT-qPCR, and are shown as log2 calibrated normalized relative quantities (CNRQ). Expression of genes involved in protein synthesis (**a**) and oxidative stress (**b**) were the highest in AT-fed lice and increased over time (24 → 48 hpi). (**c**) Expression of two genes associated with stress were highest in starved lice compared to either AT-, CO- or SK-fed lice. Significance was identified by two-way ANOVA (*p* < 0.05) followed by *post-hoc* Tukey test to determine pairwise significance. Within a time point, lower-case letters denote differences between groups where groups that do not share a letter are significantly different. Asterisks denote differences within a group between time points

To confirm findings from microarray analyses, transcript abundance was analyzed for a sub-set of DEGs using RT-qPCR. Genes with potential relation to feeding and energy production in *L. salmonis* were chosen for validation. There was high correlation between the RT-qPCR and microarray data (*p* < 0.05, *n* = 14 gene comparisons, Additional file 6: Table S3). Additionally, temporal trends observed in the genes from Atlantic-fed lice by microarray analysis were also detected by RT-qPCR analysis which showed overexpression of proteases (*cathepsin L, trypsin-1, neprilysin-1;* Fig. 5a), mitochondrial enzymes (*cytochrome B, cytochrome C oxidase subunit 2*; Fig. 5b), ribosomal proteins (*ribosomal protein P2, ribosomal protein 60S;* Fig. 6a), and oxidative stress-associated genes (*ferritin, high affinity copper uptake protein* 11; Fig. 6b). In contrast, in the RT-qPCR results, the overexpression of stress-associated genes (*programmed cell death 4, T-complex protein 1;* Fig. 6c) was specific to starved lice.

## Discussion

This study tested the hypothesis that transcriptomic responses of adult female *L. salmonis* on susceptible salmon are enhanced relative to the responses measured on resistant salmon. Furthermore, we predicted that characteristics of the enhanced response would be consistent with parasite fitness. Compared to Atlantic Salmon-fed lice, differential expression of genes in lice feeding on either the susceptible or resistant Pacific salmon species was relatively weak with low magnitude fold-changes and sparsely populated Gene Ontology categories. Moreover, in lice feeding on the Pacific salmonids, there was no increase in the response over time and often gene expression profiles were more similar to those observed in starved *L. salmonis* (e.g., *heat shock protein 90*, *tubulin alpha chain*, and *T-complex protein 1*). The similarity of transcriptional responses in the Pacific salmon-fed lice, despite differences in natural resistance of Coho and Sockeye Salmon to *L. salmonis*, indicates that host resistance status does not explain the differential parasite response. We considered the possibility that the enhanced transcriptomic response in lice feeding on Atlantic Salmon was because of host acclimation as the lice were originally collected from Atlantic Salmon [31]. This possibility was addressed by conducting a reciprocal host exposure study using lice collected from Sockeye Salmon or Atlantic Salmon. In the event of acclimation, we expected an enhanced parasite response when lice were allowed to feed on the species from which they had been collected. However, we consistently observed that lice responded strongly to Atlantic Salmon, irrespective of the source host species, thus corroborating the host-effect hypothesis and further illustrating the desirability of Atlantic Salmon to the Pacific salmon louse.

The characteristics of the transcriptomic response to Atlantic Salmon suggest increased parasite fitness. Host blood is a main dietary component of the adult female salmon louse [32] suggesting a need for haemolytic enzymes and anti-coagulants to maintain a free flow of blood to the site of feeding as observed for other hematophagous parasites [33–37]. Our data show that proteolytic and other digestive-associated enzymes were a major component of the salmon louse response to Atlantic Salmon (Table 1). The overexpression of *cathepsin L*, *trypsin-1*, *neprilysin-1*, *carboxypeptidase B*, *zinc carboxypeptidase A1*, and *legumain* was more pronounced on Atlantic-fed *L. salmonis*, consistent with earlier reports of the secretion of proteases by this parasite [38–40]. Many of these genes are known virulence factors in other ectoparasites, and modulate the host immune response during feeding: cathepsin L is a virulence factor found in numerous parasites [41–43] that suppresses the host immune response while aiding in tissue digestion [42, 44]; legumain is important in blood digestion [41]; neprilysin is associated with regulating inflammation [45]; and carboxypeptidase-B prevents clotting and inhibits inflammation [46–48]. These virulence factors likely provide similar functions for *L. salmonis*.

We detected the expression of several other feeding-associated genes in *L. salmonis* that may represent virulence factors based on functions observed in other organisms. For example *phospholipase A2* (*PLA2*) associated with feeding on Atlantic Salmon, is an important constituent of bee and snake venom [49, 50] and is also found in the secretions of hematophagous ectoparasites [49]. Interestingly, *PLA2* induces a type-2 immune response in mice [50] and possesses potent hemolytic activity [37]. Another potential virulence factor detected in feeding lice was *L-amino acid oxidase*, a toxin found in snake venoms [51]. At 48 hpi, this gene was overexpressed in feeding *L. salmonis* irrespective of the host species. Finally, a *saposin-B like* protein was significantly overexpressed in the feeding salmon louse transcriptome, and most highly by parasites feeding on Atlantic Salmon. Saposin-like proteins (SAPLIPs) have been described from *Fasciola* spp. [52], *Schistosoma mansoni* [53], *Entamoeba histolytica* [54], and *Amblyomma americanum* [55] and are involved in cytolysis and lipid metabolism [56]. The high abundance of unique contigs containing the saposin-like protein domain indicates a need to further characterize the function of SAPLIPs in *L. salmonis*. Similarly, the potential for hemorrhagic, fibrinolytic, cytolytic, and apoptotic effects, among others, warrants further investigation of the role of *L-amino acid oxidase* and *PLA2* in the salmon louse.

Genes related to oxidative stress and iron homeostasis (e.g., *ferritin*, *high-affinity copper-uptake protein-1*) were up-regulated in feeding *L. salmonis*, and expression was highest on Atlantic Salmon. In crustaceans, copper is critical as a cofactor for enzymes involved in many physiological processes including oxidative phosphorylation and mobilization of iron [57, 58]. The mitochondrial enzyme cytochrome c oxidase (cox) is a particularly abundant cuproprotein [59]. We observed prominent overexpression of metabolism-associated genes including *cytochrome c oxidase* (*subunit 2* and *3*) in lice feeding on Atlantic Salmon. Enhanced mitochondrial activity, combined with significant overexpression of protein synthesis-related genes (e.g., *ribosomal protein P2, 60S ribosomal subunit*), indicate that feeding on Atlantic Salmon was associated with pronounced metabolic activity.

Enhanced reproductive output is another proxy for higher parasite fitness, which influences host-choice and parasite virulence [2]. The salmon louse has been shown to prefer Atlantic Salmon, and parasite reproductive output and growth rates are increased while parasitizing Atlantic Salmon compared to Coho Salmon [6, 60]. We provide evidence for enrichment of reproductive-associated transcripts (e.g., *placental protein-11, neutral ceramidase, granulin-7*) in *L. salmonis* feeding on Atlantic Salmon. Further to this, enrichment for reproduction-like pathways was only present in Atlantic-fed lice compared to either Coho- or Sockeye-fed lice.

We show distinct responses to Atlantic and Pacific salmonids, possibly reflecting alternative mechanisms by which the parasite contributes to the outcome of infection. The enhanced exploitation of Atlantic Salmon may be due to differences in host-specific factors such as skin structure (e.g., low mucous cell density [61]) or physiology (e.g., low immune response [62]), or due to reduced genetic diversity in farmed populations [63, 64]. Furthermore, coevolution of the Pacific louse subspecies with Pacific Salmon for between 4.6 and 11 Ma [65] may have led to adaptations in the host-parasite relationships including variations in natural host resistance among salmon species, similar to what is observed in other host-parasite systems [66], and possibly related to host life history strategies [67–69] and life stages [70]. For example, juvenile Chum and Sockeye Salmon support high infections with *L. salmonis* and exhibit weakened cellular and humoral inflammatory responses at the louse attachment site, compared to those of juvenile Coho or Pink Salmon [15–18, 60]. Coho Salmon also exhibit heightened resistance towards other ectoparasitic copepods [4]. Similarly, juvenile Pink Salmon display a resistant phenotype that results in rapid rejection of the parasite [17, 18, 71, 72]. However mature Pink Salmon lose much of this natural resistance [70], and large abundances of the parasite are observed on mature Pink Salmon [73].

Our data support the hypothesis that Atlantic Salmon provides a host environment more permissive for *L. salmonis* fitness as shown by overexpression of transcripts related to virulence factors, energy metabolism, and reproduction. Functional enrichment for reproduction was observed during louse infection of Atlantic relative to either Coho or Sockeye. High energy metabolism is correlated with high reproductive output, and as such parasites have evolved to prefer hosts in which they are able to extract the highest available energy and maximize reproductive output [74]. Energy metabolism and reproductive output may be used as a proxy for the relative nutritional value of the host and our data suggest the nutritional gain from Atlantic Salmon exceeded that from Coho or Sockeye Salmon. Balancing host immunity with nutritive value is a driver of parasite host-specificity observed in many host-parasite relationships [75, 76], and may explain the observed host preference of *L. salmonis* for Atlantic Salmon.

## Conclusions

Understanding host-specific feeding responses of *L. salmonis* may help explain the variable outcomes of infection among host species that have previously been associated with diverse host responses, such as the delayed or muted inflammation in Atlantic, Chum and Sockeye Salmon [13, 15–19, 77]. Contrary to our original expectations, the responses were similar for parasites feeding on Coho or Sockeye Salmon*,* despite differences in their susceptibility, but differed strongly with those of lice feeding on Atlantic salmon. We suggest that the prolonged evolutionary relationship between *L. salmonis oncorhynchi* and *Oncorhynchus* spp. explains the more limited capacity for parasite response. In contrast, the non-native Atlantic Salmon elicits an enhanced parasite feeding response, which may dampen local host response mechanisms and facilitate an environment more conducive to parasite fitness. The identification of host-specific factors involved in enhanced functional and transcriptomic lice responses (e.g. constituents of mucous) will further improve our knowledge on this system. Furthermore, a comparison of the responses of the Atlantic salmon louse (*L. salmonis salmonis*) on Pacific and Atlantic salmonids will be valuable to confirm the role of co-evolutionary host-parasite interactions in enhanced parasite fitness.

## Methods

### Experimental fish and infection procedures

#### Host-effect hypothesis

Atlantic Salmon parr were obtained from a commercial salmonid hatchery, Coho Salmon parr were obtained from the Chase River hatchery on Vancouver Island, British Columbia (B.C.), Canada and Sockeye Salmon parr were obtained from the Inch Creek hatchery, Chilliwack, B.C., as previously described [15]. All fish were reared in brackish water (~15 ppt) until smoltification, after which they were maintained on ultraviolet-treated salt water (~33 ppt) in single-pass flow-through tanks and on a 12:12 h light:dark cycle. Fish were fed 1% total biomass daily with a commercially available diet (EWOS). Fish of each species were randomly divided among 12 tanks (330 L), with four tanks used for each species (2X infection tanks, 2X control tanks). Fish were acclimated for approximately 7 days and starved at least 24 h prior to any manipulation as previously described [15].

All experiments utilized the Pacific salmon louse, *Lepeophtheirus salmonis oncorhynchi*, referred to here as *L. salmonis*. Adult female *L. salmonis* were collected during harvest of Atlantic Salmon at a commercial aquaculture site on Vancouver Island, B.C. After collection, the lice were rinsed in fresh seawater and transported to the Pacific Biological Station, Nanaimo, B.C. in 8 °C aerated previously-sterilised sea water. Only lice that were firmly attached to the collection container were included in the study. The time between collection of the salmon lice and initial infection was < 24 h [15].

For experimental infections, the water level of each tank was reduced by 50%, and fish (*n* = 25/species) were sedated in seawater containing 0.2 mg/L metomidate hydrochloride (M-HCl; Aquacalm, Syndel Laboratories). Sedated fish were transferred to a temporary tank containing M-HCl (0.2 mg/L) to which 5 lice/fish were added and allowed to settle and attach [15]. Once infected with 5 lice, fish were gently removed from the infection tank and returned to their original tank. Another group of lice were maintained at 8 °C in aerated seawater and thus served as non-attached controls.

At both 24 and 48 h post-infection (hpi), one louse was sampled from each of ten Atlantic (mean weight 218 ± 29 g), Coho (mean weight 192 ± 35 g) and Sockeye Salmon (mean weight 167 ± 17 g). These 60 lice comprised the feeding lice and a further 20 lice (10 from each sample time) comprised the non-feeding controls (i.e., starved lice, Fig. 1a). Upon sampling, each louse was individually snap-frozen in liquid nitrogen for gene expression profiling.

#### Acclimation hypothesis experiment

To test the importance of the source host species on subsequent parasite responses (i.e. parasite acclimation), a second experiment was conducted using lice collected from either Atlantic or Sockeye Salmon (Fig. 1b). Adult female *L. salmonis* were collected during harvest at a commercial aquaculture site (Atlantic-acclimated; *L. salmonis*-AT) or during a test fishery (Sockeye-acclimated; *L. salmonis*-SK) and transported to the Pacific Biological Station. Salmon of each species were divided into two tanks (4 tanks; *n* = 15 per tank) and allowed to acclimate for 7 days. Infections were performed as above except that for each species, fish in one tank were infected with *L. salmonis*-AT (*n* = 5 lice per fish) and those in the second tank infected with *L. salmonis*-SK (*n* = 5 lice per fish). At 24 and 48 hpi, 8 lice were collected from each tank (total number of lice = 32 per time) and snap-frozen in liquid nitrogen.

### RNA extraction

Frozen lice were homogenized using 5 mm stainless-steel beads and a Tissue-lyser (Qiagen). RNA was extracted using TRIzol (Invitrogen) following manufacturer’s instructions with modifications. Specifically, following the organic phase extraction, the supernatant was removed and RNA was then purified using RNeasy spin columns (Qiagen) with an on-column DNase I digestion to remove genomic DNA as per manufacturers’ instruction. Total RNA was eluted in 30 μL ultra-pure water and quantified by spectrophotometry (Nanodrop-1000, Thermo Fisher). RNA quality was determined using Experion Automated Electrophoresis (Bio-Rad) with all samples having an RQI < 9.

### cRNA synthesis and reference pool generation

Purified total RNA (200 ng) was reverse-transcribed to cDNA and then amplified to Cy5-labeled cRNA with Cy5-CTP-labeled nucleotides (Perkin Elmer) as previously described [78] using Low Input Quick Amp Labeling kits (Agilent), as per manufacturer’s instructions for hybridization to a 4-pack oligo gene expression microarray. Labelled cRNA was purified through RNeasy columns as per manufacturer’s instructions (QIAGEN) and quantified using spectrophotometry (NanoDrop-1000), ensuring specific activity of all samples > 6 pmol dye per microgram cRNA (Agilent). Samples were kept at −80 °C until hybridization. A reference pool of Cy3-cRNA was synthesized by amplifying experimental samples as described above, but with Cy3-CTP-labeled nucleotides (Perkin Elmer). In each experiment, a reference pool of equimolar cRNA was generated from each experimental condition (*n* = 10 individuals).

### Microarray hybridization, quantification, normalization and filtering

A 38 K oligo microarray was designed using previously annotated ESTs from both Pacific and Atlantic *L. salmonis* [28] using eArray (Agilent). Probes were preferentially selected at 3′ untranslated regions and each EST was represented by duplicate probes (19 k ESTs represented on the array). Each individual louse was hybridized to a single array (i.e. total of 20, 4-pack microarrays hybridized in the host-effect experiment). Sample and reference combinations (825 ng cRNA each) were fragmented and then hybridized at 65 °C for 17 h at 10 rpm as per manufacturer’s instructions (Agilent) using SureHyb chambers (Agilent). Washing was performed as per manufacturers’ instructions, using the optional protocol to prevent ozone degradation. All slides were transferred to a dark box and kept at low ozone until scanned on a Perkin Elmer ScanArray® Express at 5 μm resolution using PMT settings optimized to have the median signal of ~1–2% of array spots saturated (Cy5: 65; Cy3: 68). Spot intensities were quantified in Imagene 8.1 (Biodiscovery) using an eArray GAL file (Design ID: 024389; Agilent). Poor spots and control spots were flagged by the software for downstream filtering. The background of each spot was subtracted from the foreground median, and samples were imported into GeneSpring 11.5.1 (Build 138755; Agilent). Each experiment was normalized and filtered separately as follows: raw value threshold of 1.0; intensity-dependent *Lowess* normalization; and baseline transformation to the median of all samples. Control spots and any probes not passing the following filter were removed from the analysis: raw values ≤ 500 in at least 65% of samples in any one condition and no flags in at least 65% of samples in any one condition as described elsewhere [78]. Raw quantified microarray files have been submitted to NCBI Gene Expression Omnibus (GEO) under the accession GSE80220.

### Differential expression and functional analysis of microarray data

Probes were tested for differential expression using a two-way ANOVA without equal variance assumption using host-species and time as explanatory variables, followed by a post hoc Tukey’s HSD (*p* < 0.01) and using an FDR (Bonferroni’s test for multiple test correction) and fold change ≤ 1.5 from starved controls. All differentially expressed probes were used as an input for *k*-means clustering to identify co-expressed gene clusters (Euclidean distance metric; 5 clusters; 50 iterations; GeneSpring 11.5.1 Agilent). Gene Ontology (GO) and pathway enrichment were performed on annotated probes using the DAVID online bioinformatics tool (modified Fisher’s exact test) [79], with UniProt accessions of clustered probes compared to a background list of all probes passing quality control filters (n = 15,718 probes). Overlap between lists of differentially expressed genes was evaluated using *VENNY* [80]. After Gene Ontology enrichment analysis, GO Trimming was used to reduce redundancy of enriched Gene Ontology categories with a soft trim threshold of 40% [81].

### Reverse-transcriptase quantitative polymerase chain reaction (RT-qPCR)

The same RNA samples analyzed with microarrays in the host effect experiment were used for RT–qPCR. This included a total of ten individual lice from each of four conditions (feeding on Atlantic Salmon, Coho Salmon, Sockeye Salmon, or not-feeding; see above for more details) at 24 and 48 h, to produce a total of 80 samples. Synthesis of cDNA was performed with 2 μg of total RNA in 20 μl reactions using oligo (dT) primers and AffinityScript cDNA Synthesis kits (Agilent), as per manufacturer’s instructions. Each cDNA sample was diluted 10-fold. To generate a standard curve, one sample from each of the four conditions (starved, Atlantic-fed, Coho-fed, Sockeye-fed) was randomly selected and synthesized as described previously [15, 78]. These samples were pooled and diluted 10-fold and this pool was then used for a serial dilution (5-point, 10-fold each point) for efficiency calculation. RT- qPCR amplification was performed using Brilliant UltraFast SYBR III® (Agilent) in 20 μl reactions with 0.1 μM of each primer using the following thermal regime: 95 °C for 3 min, followed by a combined annealing and extension step of 60 °C for 40 cycles. For qPCR technical replication, each sample/gene combination was run in triplicate. Genes of interest were selected from the microarray results based on biological relevance, high significance level or presence in enriched GO categories [78]. Reference gene candidates were selected from microarray results based on stable expression across conditions, consistency across replicate spots and moderate levels of expression, as well as from previous literature [78]. Primers were designed in Primer3 [82] selecting amplicon sizes of 80–150 base pairs (Additional file 7: Table S4). Amplicons were checked for single products by melt curve analysis and were sequenced on an ABI 3130 (Applied Biosystems) to confirm identity. RT–qPCR data analysis was performed using qBase-PLUS (Biogazelle). Stability of reference genes was tested using geNorm [83]. Selected reference genes included the previously identified *structural ribosomal protein S20* and *tubulin beta chain*, with a collective M value of 0.382 and CV of 0.146, which is within the range typically observed for stably expressed reference genes in heterogeneous sample [83]. Other tested reference genes that were not used to normalize due to higher variability included *elongation factor 1-α* and *HPGRT*. NTC and RT controls showed no amplification. Statistical significance was identified by two-way ANOVA (*p* < 0.05) with pairwise significance determined by post-hoc Tukey test (SigmaPlot V11.1). Correlation between methods (RT–qPCR and array) was tested using by the correlation of log_2_ normalized expression values for RT–qPCR samples against microarray normalized log_2_ expression ratios (Cy5/Cy3) for the probe corresponding to the contig used for primer design, as previously described (Additional file 6: Table S3; [18]).

## Additional files

Additional file 1: Table S1. Numbers of differentially expressed genes detected in *L. salmonis* while feeding on different host species (i) compared to starved lice, or (ii) compared to each other. (XLSX 25 kb)
Additional file 2: Transcriptomic response of *L. salmonis* while feeding on Atlantic, Coho or Sockeye Salmon. (XLSX 429 kb)
Additional file 3: Figure S1. Profiling the starvation response of *L. salmonis.* Overexpressed transcripts in lice withheld from hosts were compared to Atlantic-, Coho- or Sockeye-fed lice to produce a list of unique genes involved in the “starvation response” of *L. salmonis*. These genes were analyzed using DAVID to produce enriched gene lists after (A) 24 and (B) 48 hpi. ^a^Fold Enrichment. (TIF 14475 kb)
Additional file 4: Table S2. Gene Ontology enrichment of the host-specific feeding response in *L. salmonis*. Functional enrichment of DEGs from Atlantic-fed compared to either Coho-fed or Sockeye-fed lice at 24 and 48 hpi, and from either Coho-fed or Sockeye-fed lice compared to Atlantic-fed lice at 24 hpi. (XLSX 11 kb)
Additional file 5: Figure S2. The salmon louse response cannot be explained by acclimation to the host. A reciprocal experiment was conducted by placing lice originally collected from Atlantic (at) or Sockeye Salmon (sk) on either Atlantic Salmon (AT) or Sockeye Salmon (SK) hosts. Expression of protein synthesis (A), energy metabolism (B) and digestion (C) was significantly higher in lice feeding on Atlantic Salmon (blue boxplots) irrespective of their original host, thus negating the acclimation hypothesis. Differentially expressed transcripts identified by the microarray were profiled using RT-qPCR, and are shown as log2 calibrated normalized relative quantities (CNRQ). Significance was identified by two-way ANOVA (*p* < 0.05) followed by *post-hoc* Tukey test to determine pairwise significance. Differences between groups are denoted by lower case letters, while differences over time within a group is denoted by an asterisk (*). (TIF 17046 kb)
Additional file 6: Table S3. Validation of microarray experiments. The expression profiles of a select subset of genes were compared between RT-qPCR and microarray results. Expression levels were validated by testing the correlation of log2 expression values for RT–qPCR samples against microarray log2 expression ratios (Cy5/Cy3). (XLSX 75 kb)
Additional file 7: Table S4. List of primer sequences for RT-qPCR. (XLSX 42 kb)

## Abbreviations

CDD: Conserved domain database
FC: Fold change
GEO: Gene expression omnibus
GO: Gene ontology
