# Supplementary figures and images for: Enhanced transcriptomic responses in the Pacific salmon louse Lepeophtheirus salmonis oncorhynchi to the non-native Atlantic Salmon Salmo salar suggests increased parasite fitness
Source: BMC Genomics. 2017 Jan 30;18:110. doi: 10.1186/s12864-017-3520-1 (PMC5282744; doi:10.1186/s12864-017-3520-1)

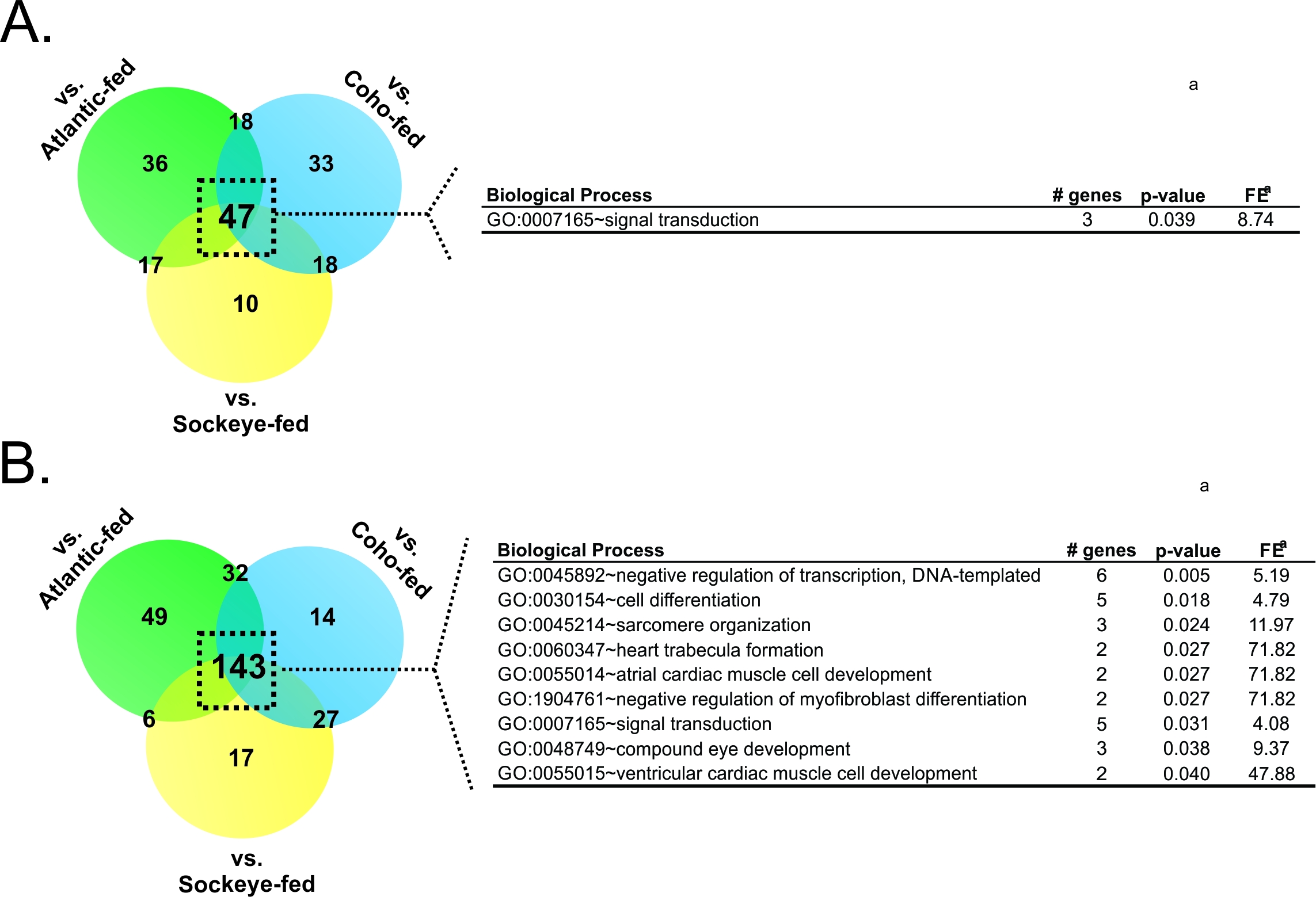

Supplement: Additional file 3: Figure S1. — Profiling the starvation response of L. salmonis. Overexpressed transcripts in lice withheld from hosts were compared to Atlantic-, Coho- or Sockeye-fed lice to produce a list of unique genes involved in the “starvation response” of L. salmonis. These genes were analyzed using DAVID to produce enriched gene lists after (A) 24 and (B) 48 hpi. aFold Enrichment. (TIF 14475 kb) [file 12864_2017_3520_MOESM3_ESM.tif]

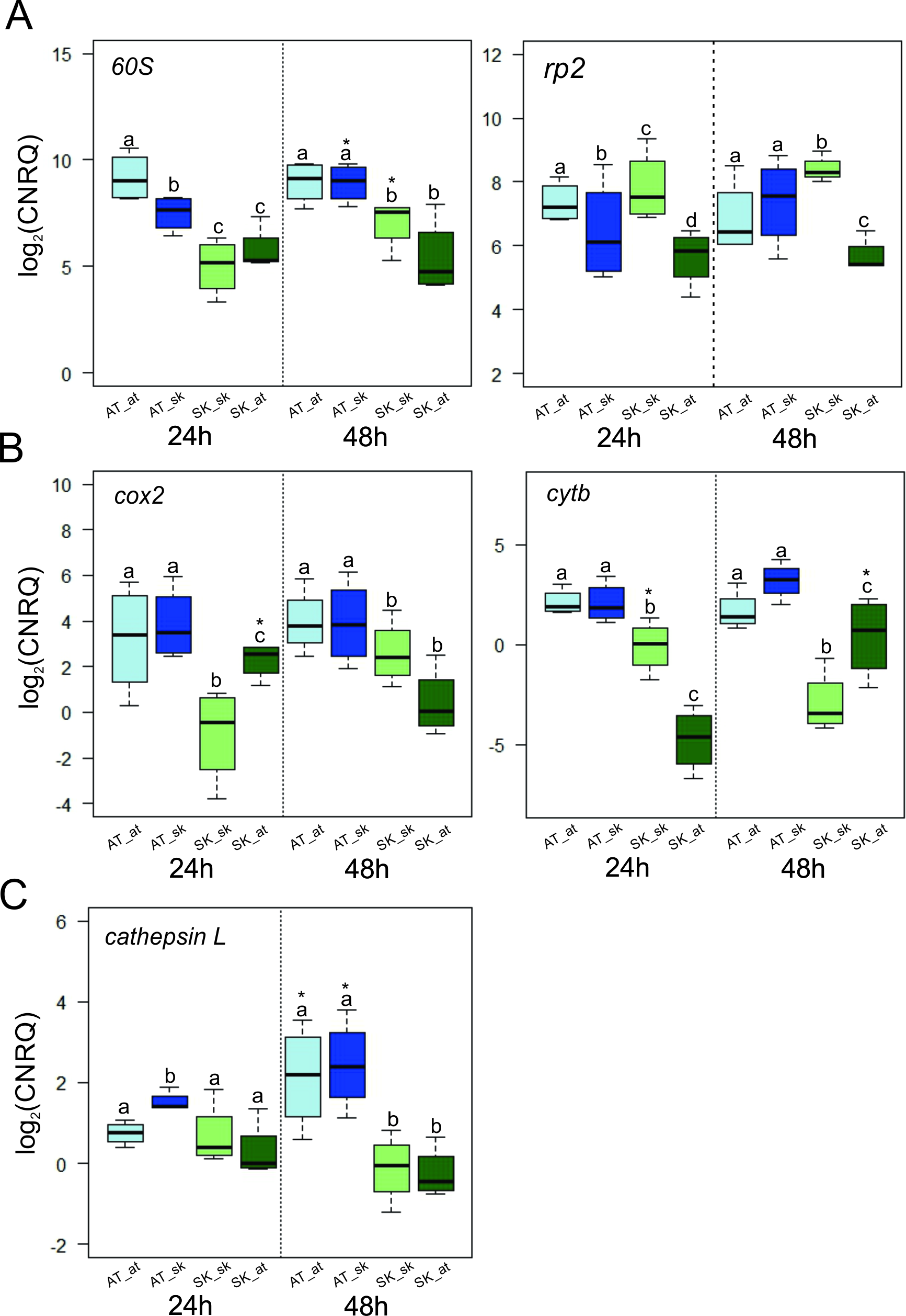

Supplement: Additional file 5: Figure S2. — The salmon louse response cannot be explained by acclimation to the host. A reciprocal experiment was conducted by placing lice originally collected from Atlantic (at) or Sockeye Salmon (sk) on either Atlantic Salmon (AT) or Sockeye Salmon (SK) hosts. Expression of protein synthesis (A), energy metabolism (B) and digestion (C) was significantly higher in lice feeding on Atlantic Salmon (blue boxplots) irrespective of their original host, thus negating the acclimation hypothesis. Differentially expressed transcripts identified by the microarray were profiled using RT-qPCR, and are shown as log2 calibrated normalized relative quantities (CNRQ). Significance was identified by two-way ANOVA (p < 0.05) followed by post-hoc Tukey test to determine pairwise significance. Differences between groups are denoted by lower case letters, while differences over time within a group is denoted by an asterisk (*). (TIF 17046 kb) [file 12864_2017_3520_MOESM5_ESM.tif]
